# How social/environmental determinants and inflammation affect salivary telomere length among middle-older adults in the health and retirement study

**DOI:** 10.1038/s41598-022-12742-z

**Published:** 2022-05-25

**Authors:** Margaret Gough Courtney, Josephine Roberts, Kanya Godde

**Affiliations:** University of La Verne, 1950 Third St., La Verne, CA USA

**Keywords:** Epigenetics, Anatomy, Biomarkers

## Abstract

Social epidemiology posits that chronic stress from social determinants will lead to a prolonged inflammatory response that may induce accelerated aging as measured, for example, through telomere length (TL). In this paper, we hypothesize variables across demographic, health-related, and contextual/environmental domains influence the body’s stress response, increase inflammation (as measured through high-sensitivity C-reactive protein (hs-CRP)), and thereby lead to shortening of telomeres. This population-based research uses data from the 2008 Health and Retirement Study on participants ages ≤ 54–95 + years, estimating logistic regression and Cox proportional hazards models of variables (with and without confounders) across the domains on shortened TL. A mediation analysis is also conducted. Contrary to expectations, hs-CRP is not associated with risk of shortened TL. Rather, factors related to accessing health care, underlying conditions of frailty, and social inequality appear to predict risk of shorter TL, and models demonstrate considerable confounding. Further, hs-CRP is not a mediator for TL. Therefore, the social determinants of health examined do not appear to follow an inflammatory pathway for shortened TL. The finding of a relationship to social determinants affecting access to health care and medical conditions underscores the need to address social determinants alongside primary care when examining health inequities.

Telomeres fulfill a number of important functions, including helping protect the genome. They naturally shorten with age, but shorter telomere length (TL) is also linked to a number of chronic older age-associated conditions^1^, including cardiovascular disease^2^, and osteoporosis^3^, among others. TL’s causal role for aging is questioned^4^, but its inverse association with mortality is well-established, although the association decreases as one ages^5^. It has been hypothesized that TL varies at birth, with some individuals being born with longer or shorter telomeres, instead of them shortening over time due to oxidative stress and inflammation^6^. Others disagree. More recently, the “accumulating costs hypothesis” proposes telomeres shorten over time as a result of moderate stressors and mild diseases over the life span^7^. Similarly, the “weathering hypothesis” has been extended to TL, stating an accumulation of population-specific social stressors leads to decreases in TL^8^. As individuals age (at a normal or accelerated pace), most will develop increased inflammation that elevates susceptibility to a number of health conditions, and ultimately leads to death in a process called “inflammageing”^9^. Inflammageing shortens telomere length^9^; as such, short TL is often considered a marker of accelerated aging that is influenced by chronic stress^10^, indicating that TL may be a mechanism through which exposure to prolonged stress leads to adverse health outcomes.

Briefly, the accumulating costs and weathering hypotheses are coupled with a social epidemiological theoretical model (based on^11–14^), which states that certain prolonged non-biological exposures can induce a stress response (acute or chronic) in the body that, when chronic, can lead to increased inflammation, and therefore physiological changes, such as shortening of telomeres (see Fig. 1, tailored to this paper). More specifically: 1. Prolonged stress exposure engages the hypothalamic–pituitary–adrenal (HPA) axis to respond; 2. Leading to increased allostatic load (a combination of acute and chronic stress responses), and 3. Subsequently higher levels of stress hormones that elevate inflammatory biomarkers^11^. The question is whether these greater levels of inflammation typically lead to shorter telomeres. To provide a concrete example of the theoretical framework, childhood adversity^15^ is a well-known example of a factor that can induce inflammatory responses and telomere shortening. Shortened TL is associated with the inflammatory biomarker high-sensitivity C-reactive protein (hs-CRP)^16^, and both with social determinants of health^17,18^, although a causal relationship has not been established^16^. In this population-based study using the Health and Retirement Study (HRS), we examine shortened TL’s association with the only inflammatory biomarker available in the only wave with TL data: hs-CRP.

**Figure 1:**
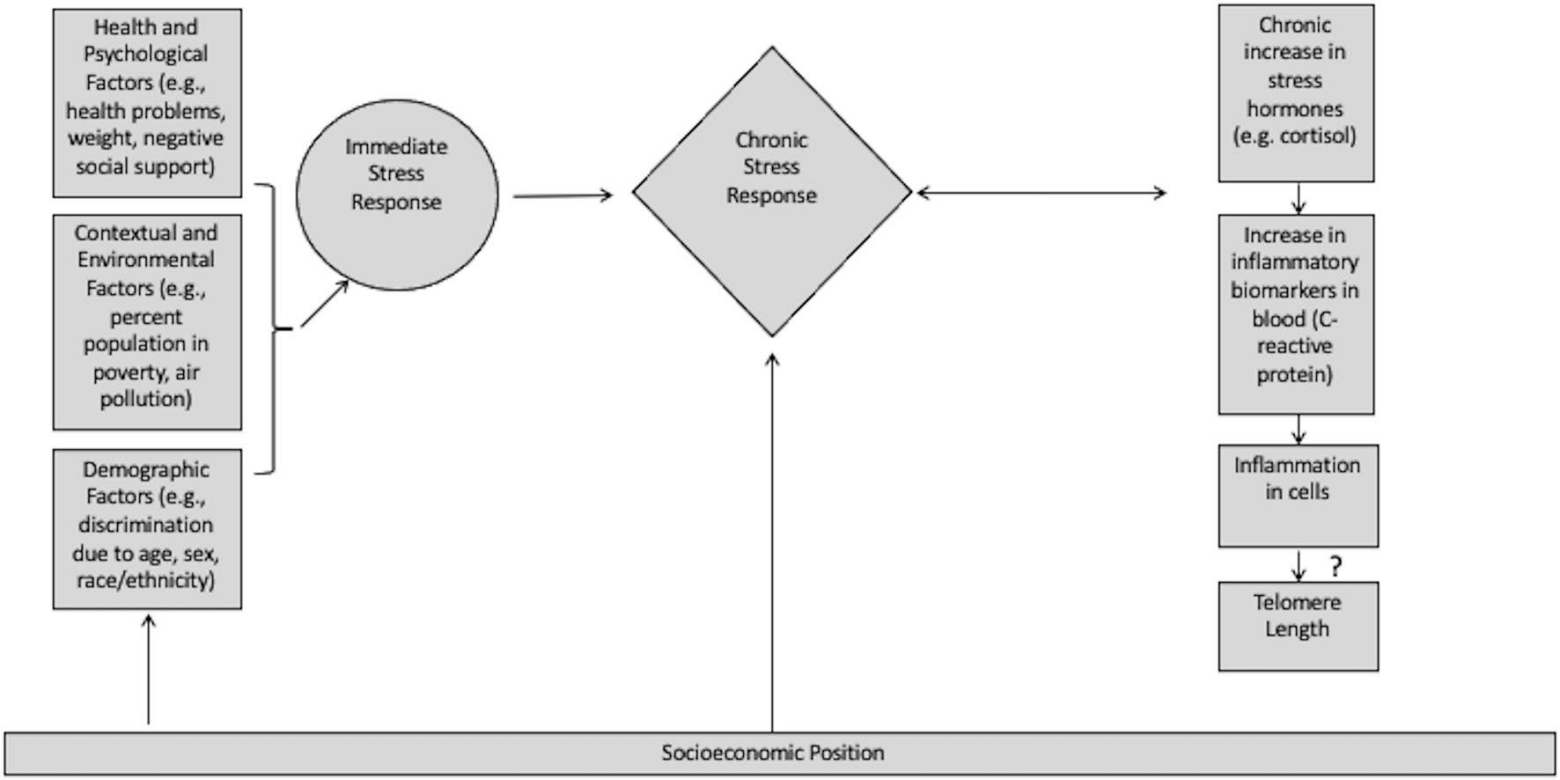
Conceptual model of social determinants on TL. Derived from Barr^11^, Kubzansky^13^, Riancho and Brennan-Olsen^14^, and Gough and Godde^12^.

As noted in the conceptual diagram (Fig. 1), there are three major domains of interest in this paper. These domains, and the factors within them, were identified through variable selection (see Materials and Methods) that narrowed six original domains of interest (demographic, social, economic, health-related, political, contextual/environmental) drawn from the literature down to three (demographic, health-related, contextual/environmental) from which predictors and confounders were analyzed.

The rate of TL shortening varies considerably across demographic and contextual factors^1^, as individuals age and TL naturally shortens. Demographic variables associated with TL include being female (longer TL)^19^, race/ethnicity^20^, and education^21,22^. Health and psychological variables linked to TL are numerous, such as having ever smoked^23^, increased weight^24^, increased intensive care unit stays^25^, chronic periodontal disease via higher systemic inflammation and oxidative stress;^26^, increased hs-CRP (e.g.,^27^), psychosocial stress^28^, early life adversity (e.g.,^29^), and lifetime adversity^10^. Contextual and environmental factors that affect TL are limited in the literature; only four were identified, representing air and neighborhood quality: short-term exposure to PM_2.5_ (longer TL^30^), long-term PM_2.5_ exposure (shorter TL^18^), ozone exposure in chronically ill patients (longer TL^30^), and poor perceived neighborhood quality (shortened TL^31^).

Although there is a great interest in understanding the social determinants of chronic stress and subsequently, shortened TL, few studies have examined a wide range of potential predictors in the same analysis. In this study, we advance the literature by providing a comprehensive analysis of demographic; health- and psychological-related; and contextual and environmental factors that may influence TL using data from the HRS. Using a cross-sectional design, we seek to differentiate between significant predictors and confounders that are attributed as risk factors for shortened TL in the literature, understanding we are only testing whether individuals with increased stress also have shorter telomere lengths, since we do not have repeated observations. Consistent with the social epidemiological theoretical model described earlier, we hypothesize that demographic, health and psychological, and contextual and environmental exposures predict a chronic stress response in the body that leads to inflammation and shortened telomeres. As the literature is mixed as to a causal role of telomere length, we assess this hypothesis holistically using models with and without a measure of inflammation that has been linked to TL and social determinants—hs-CRP—to further understand whether hs-CRP is a mechanism through which chronic exposure to stressors results in shorter TL. We provide models without confounders to measure the unbiased odds and hazard ratios and a mediation analysis to further evaluate the role of hs-CRP.

## Method

### Data

The data for this population-based study come from the 2008 wave of the HRS, the only wave in which telomere data are available. The HRS was initiated in 1992 as the first nationally representative longitudinal study of community-dwelling older adults that included both economic and health information. We combine data from the RAND Longitudinal File^32^ with data from the Cross-Wave Race and Ethnicity File^33^, Psychosocial and Lifestyle Questionnaire (part of the Core survey)^34^, sensitive health (and therefore, restricted-use) 2008 Biomarker Study^35^, 2008 Telomere Data Study^36^, HRS-Contextual Data Resource (CDR)^37^, and The Correlates of State Policy Project^38^. The RAND Longitudinal file^32^ includes cleaned and processed variables from the Core and Exit Interviews^34^ of the HRS. The 2008 Biomarker Study^35^ and 2008 Telomere Study^36^ include roughly half of the sample who was assigned to an Enhanced Face-to-Face interview at that wave. Because only half of the sample participants were in the Biomarker Study every other wave, the first wave of Biomarker Study data available for the Telomere Study participants is 2008. The HRS-CDR includes contextual data from a wide variety of sources, including the American Community Survey ACS;^37^, and EPA Air Pollution components^39,40^. The sample includes 3,761–4,853 respondents ages ≤ 54–95 + years (see Figure S1). We are unable to provide an exact age range as the cell sizes include fewer than three individuals in the minimum and maximum, which would violate the anonymity of the respondents in the restricted-use, sensitive health data for TL (for more information on data use restrictions and agreements see^41^). However, researchers who gain access to the data can replicate our exact sampling with the information provided in the Method section. Our secondary data study was deemed exempt by the University of La Verne Institutional Review Board (Protocol Number: 2019–13-CAS). Further, all analyses in this research were performed in accordance with the relevant guidelines and regulations, as well as by the researchers who compiled the HRS^41^.

### Variables

The dependent variable is salivary telomere length, obtained from the 2008 Telomere Study. The assays were completed by Telomere Health via quantitative PCR (qPCR) by creating a T/S ratio analogous to mean telomere length from the telomere sequence copy number (T) and the single-copy gene copy number (S)^36^. The construction of the variable for this paper followed the protocol in Puterman et al.^10^ as they identified three important reasons for doing so: 1) consistency with other large epidemiological cohort studies, 2) values in the lowest decile have reduced sensitivity and specificity with the methodology of measuring TL used in HRS, and 3) non-normality in the sample (an issue also detected in this research by a Shapiro–Wilk test and various attempts at achieving normality were not successful). Individuals with values greater than 2.0 were eliminated, as larger values are likely inaccurate. TL was then dichotomized such that the lowest 25% of the distribution is coded as 1 (for shortened TL), and the remainder of the distribution is coded as 0 (for normal TL).

An initial set of variables were selected from the datasets using social epidemiological theory and published articles. To narrow the dataset, we use a combination of Spearman’s correlation and change-in-estimate variable selection methods^42^ to identify important significant and confounder independent variables, using age (in the logit model) and sex (in the Cox model) as anchor variables. The anchor variables are the coefficients of interest, and in the change-in-estimate method, each additional covariate is added to the model and retained if significant or if it impacts the anchor coefficient by 10% or more^42^. The non-significant variables retained are likely confounders, and the method’s performance in identifying these confounders is excellent^42^.

The main demographic factors retained from variable selection include age, measured in years, sex (with male as reference category), and race/ethnicity. Race/ethnicity, unfortunately, only has three possible categories due to sample size: White/European American, Black/African American, and Another Race/Ethnicity. Respondent’s education level (less than high school, GED, high school graduate, some college, and college graduate), the number of children ever born (as an integer), and household income (continuous) are also incorporated into the models.

We identified several health-related and psychological measures during variable selection: self-rated health (Likert scale; continuous); weight (in kilograms) (continuous); dichotomous measures of having ever smoked, having heart problems, and having arthritis; a tripartite measure of ever having a stroke or TIA/possible stroke; and NHANES equivalent measures of high-density lipoprotein (HDL) (mg/dL), cholesterol (mg/dL) (continuous) and cystatin C (mg/L) (continuous), which were the only components of allostatic load identified during variable selection. A nine-component allostatic load index was evaluated, but it did not impact the predictor variable of interest appreciably and therefore was not included in the final model. Further, we incorporated an indicator of whether the respondent was covered by Medicare; the number of times the respondent spent the night in the hospital and the number of nursing home stays (continuous); categorical indicators of whether the respondent has seen a dentist (due to periodontal disease’s relationship to increased inflammation, oxidative stress, and shortened TL) or used home health services in the prior two years; the index of fine motor skills (continuous); and difficulty with walking one block (categorical: no; yes, a little; yes, a lot).

Four psychological measures (continuous) were also identified as important during variable selection: (1) the index of lifetime traumas before age 18 to capture childhood stressors and adversity, (2) the index of stressful life events to capture adulthood stressors and adversity (measured over the prior 5 years), and the stress of negative family (3) and friend (4) social support (Psychosocial and Lifestyle Questionnaire^43^; see Supplementary Information for a breakdown of the indices). As social support is an important mediator in the literature, and to ensure the role of positive social support was truly not important in explaining TL, we re-screened our final models of demographic and health variables, but found further evidence of social support being a confounder, so those results are not reported. Models 2, 3, 6 and 7 also add categorical measures of hs-CRP (mg/dL), the only inflammatory biomarker available from the same year of data when TL was collected: a binary measure of high hs-CRP (≤ 3 vs. > 3 and < 10^44^) in Models 2 and 6, and a tri-level measure of hs-CRP (< 1, 1–3, > 3 and < 10^17^) in Models 3 and 7.

The demographic makeup of one’s location of residence can be an important predictor of health outcomes as it relates to structural racism and structural inequality, though it has not been studied for TL. Therefore, it was no surprise that variable selection detected many contextual indicators as being important to predicting TL: the percent non-Hispanic White, percent without a high school degree of population aged 25 + , percent in poverty, percent with a high school diploma, and population density per square mile in the respondent’s county (from HRS-CDR ACS). At the state level the percent of individuals identifying as Hispanic or Latina/o is included. Similarly, the categorical individual health rate review variable (categories: file-use or no review, prior approval needed only for some products/companies, prior approval or strict medical loss ratio requirements) from the political data^38^ is used to examine health policy for the state. Measures of air pollution exposure (continuous; obtained from the HRS-CDR EPA) were also identified: mean NO_2_ (µg/m^3^), mean ozone (µg/m^3^), and mean PM_2.5_ (µg/m^3^). Although the contextual data is collected at a higher level of aggregation, our focus here is on assigning place-based characteristics to individuals, rather than examining individuals as nested within counties or states. As such, the survey weighting procedure explained in the Analytic Strategy (below) is enough to address the fact the sample was collected from strata and PSUs within the United States, and as also implemented in another recent study using HRS^45^.

### Analytic strategy

We estimate eight models. First, logistic regression models were estimated. Model 1 includes age and other variables as predictors to produce odds ratios of short TL. To look at the relationship with inflammation, in Model 2 binary hs-CRP is added to Model 1, and, in Model 3, tri-level hs-CRP is substituted for binary hs-CRP. Model 4 drops the non-significant predictors to estimate unbiased odds ratios. The models’ ability to describe the data is evaluated with McFadden’s pseudo R^2^.

Second, to look at age-dependent covariate information for TL (Model 5), a semi-parametric Cox proportional hazards model is employed with age at data collection as the time scale variable. As a survival analysis, it produces a cumulative hazard ratio of having shortened TL at a specific age (time *t*) and is specified by the equation:

1 $$h\left( t \right) = h\left( {t_{0} } \right)\exp (b_{1} X_{1} + b_{2} X_{2} + \cdots b_{p} X_{p} )$$

where *h(t)* is the hazard at some age (*t*) and the baseline (birth) is *h(t*_0_). Cox regression models on cross-sectional genetic data have been shown to have increased power compared to logistic regressions and to be an appropriate technique for analyzing this type of data^46^. The proportionality assumption for the covariates over time was tested using Schoenfeld residuals. Models 6 and 7 add the binary and tri-level measures of hs-CRP to examine the role of inflammation on TL. Model 8 drops the non-significant predictors to estimate unbiased hazard ratios. VIF tests checked for issues with multicollinearity in the models.

Third, the accuracy of the telomere models is tested using cross-validation with k-Nearest Neighbors Discriminant Analysis (k-NNDA; k = 3) in SAS^47^. The nonparametric procedure examines neighboring observations and sorts into clusters based on similarity. The algorithm uses Mahalanobis distances derived from pooled covariances.

Fourth, formal mediation assessment was conducted to further examine the potential mediating effects of hs-CRP in models without the confounding variables. This helps to identify whether hs-CRP is a mechanism in the relationship between exposure to stressors and TL. We employ the Baron-Kenny method^48^, which has four steps: 1. Use model predictors to estimate TL and look for coefficients significantly different from zero; 2. Use model predictors to estimate hs-CRP and look for coefficients significantly different from zero; 3. Use hs-CRP to predict TL, controlling for model predictors and look for a hs-CRP coefficient significantly different from zero and reduction in other predictor coefficients; 4. Estimate the relationship between TL and model predictors, accounting for hs-CRP and look for a non-significant relationship and coefficients close to zero.

The HRS is a complex sample survey, and we account for these features in our models using the *svy* commands in Stata 16.0 and the survey package^49^ in R^50^ that uses Horowitz-Thompson robust errors (sandwich standard errors) to address the minimal non-linearity of the continuous predictors to log odds present in these models. The weight used is the biomarker weight, and sampling strata and PSU information come from the RAND Longitudinal File. All analyses are judged at a = 0.05 (two-tailed). For variables with small amounts of missing data (no more than 2% of observations in the Cox model and 2–13% in the logit model) listwise deletion is employed. Comparisons of the full sample before listwise deletion and the analytic samples after listwise deletion are shown in Supplementary Tables 1 (logit) and 2 (Cox); the characteristics of the full and analytic samples are nearly identical. Further, *t*-tests and Wald tests by inflammation (binary hs-CRP) were performed on the variables in Table 1 and are presented in Supplementary Table 3.

**Table 1:** Survey-weighted descriptive statistics for analytic sample.

Variable	Without hs-CRP	With hs-CRP
	Mean (SE)/proportion	Mean (SE)/proportion
Short telomere length	0.24	0.24
Age	67 (0.21)	67 (0.22)
Sex—female	0.54	0.54
**Race/ethnicity**		
Black/African American	0.08	0.08
Another race/ethnicity	0.04	0.04
Self-reported health	2.8 (0.03)	2.7 (0.03)
Ever smoked—yes	0.57	0.57
Percent non-Hispanic White (Tract)	0.71 (0.01)	0.71 (0.01)
Covered by Medicare—yes	0.54	0.53
Number of times respondent spent night in the hospital	0.42 (0.02)	0.38 (0.02)
Seen dentist in previous 2 years—yes	0.68	0.69
Index of lifetime traumas before age 18	0.47 (0.02)	0.47 (0.02)
Index of stressful life events	0.34 (0.02)	0.33 (0.02)
Mean NO_2_ for September (µg/m^3^)	7.8 (0.19)	7.8 (0.19)
Mean O_3_ for April (µg/m^3^)	47 (0.15)	47 (0.16)
Mean O_3_ for May (µg/m^3^)	47 (0.25)	47 (0.25)
Mean O_3_ for November (µg/m^3^)	28 (0.22)	28 (0.22)
**High sensitivity C-reactive protein (CRPtri) (mg/dL)**		
Between 1–3		0.42
Greater than 3		0.31

*hs-CRP* high sensitivity C-reactive protein, *SE* standard error. n = 4,206 for analytical sample without hs-CRP and n = 3,761 for analytical sample with hs-CRP.

## Results

Descriptive statistics are shown in Table 1. The age range of the sample is ≤ 54–95 + years, with a mean age of about 67 years. A little over half of the sample is female. In Model 1 (Table 2), older age is associated with a higher odds of short TL, as is having ever smoked, spending more nights in the hospital, and having a higher score on the index of lifetime traumas before age 18. Being female is linked with lower odds of short TL, as is identifying as Black/African American, having seen a dentist in the past two years, and, somewhat unexpectedly, a higher score on the index of stressful life events during the prior five years. The remaining variables reported in Model 1 are confounders. The McFadden’s pseudo R^2^ of 0.05 indicates the model fits the data rather poorly, likely due to heterogeneity, but trends are identifiable. The accuracy of the model to correctly predict shortened TL is 57.56% (k-NNDA), which is also low. Although model fit is relatively poor, it is comparable to, or better than, other recent studies^51–53^.

**Table 2:** Odds ratios from logistic regression models of short telomere length.

Variable	Model 1: Without hs-CRP		Model 2: With binary hs-CRP	
	OR (95% CI)	*﻿p*	OR (95% CI)	*p*
Intercept	0.03 (0.01, 0.1)	< 0.0001*	0.03 (0.01, 0.12)	< 0.0001*
Age	1.02 (1.01, 1.03)	< 0.0001*	1.03 (1.01, 1.04)	0.0001*
Sex—female	0.82 (0.73, 0.91)	0.0352*	0.77 (0.64, 0.93)	0.0086*
**Race/ethnicity**				
Black/African American	0.56 (0.37, 0.85)	0.0095*	0.58 (0.38, 0.89)	0.0174*
Another race/ethnicity	0.77 (0.47, 1.3)	0.3069	0.78 (0.45, 1.3)	0.3648
Self-reported health	1.02 (0.93, 1.1)	0.7253	1.04 (0.95, 1.1)	0.3922
Ever smoked—yes	1.3 (1.4, 1.6)	0.0012*	1.4 (1.1, 1.6)	0.0014*
Percent non-Hispanic White	1.2 (0.87, 1.7)	0.2433	1.2 (0.84, 1.7)	0.3360
Covered by Medicare—yes	1.2 (0.95, 1.5)	0.1431	1.1 (0.86, 1.5)	0.3858
Number of times respondent spent night in the hospital	1.1 (1.0, 1.2)	0.0040*	1.2 (1.0, 1.3)	0.0062*
Seen dentist in previous 2 years—yes	0.57 (0.47, 0.7)	< 0.0001*	0.55 (0.45, 0.68)	< 0.0001*
Index of lifetime traumas before age 18	1.2 (1.1, 1.4)	0.0027*	1.2 (1.1, 1.4)	0.0038*
Index of stressful life events	0.81 (0.70, 0.92)	0.0061*	0.81 (0.69, 0.96)	0.0174*
Mean NO_2_ for September (µg/m^3^)	1.0 (0.97, 1.0)	0.8537	1.0 (0.97, 1.0)	0.9395
Mean O_3_ for April (µg/m^3^)	1.0 (0.97, 1.0)	0.9881	0.99 (0.96, 1.0)	0.7956
Mean O_3_ for May (µg/m^3^)	1.0 (0.99, 1.0)	0.2552	1.0 (0.99, 1.0)	0.2681
Mean O_3_ for November (µg/m^3^)	1.0 (0.98, 1.0)	0.8194	1.0 (0.98, 1.0)	0.9556
**High sensitivity C-reactive protein (mg/dL)**				
Greater than 3			0.91 (0.75, 1.1)	0.3441

*hs-CRP* high sensitivity C-reactive protein, *OR* odds ratio, *CI* confidence interval.

*p < 0.05 as significant findings in this analysis.

Model 2 (Table 2) results are very similar to those in Model 1, and binary hs-CRP is not a significant predictor of short TL. The McFadden’s pseudo R^2^ is still 0.05 and k-NNDA accuracy is similar at 58.55%. Finally, Model 3 (Table 3) results are largely consistent with Models 1 and 2. As in Model 2, the hs-CRP variable is not a significant predictor of TL. McFadden’s pseudo R^2^ remains 0.05, and k-NNDA accuracy is 58.36%.

**Table 3:** Odds ratios from logistic regression models of short telomere length.

Variable	Model 3: With tri-level hs-CRP		Model 4: Without Cofounders	
	OR (95% CI)	*p*	OR (95% CI)	*p*
Intercept	0.03 (0.01, 0.12)	< 0.0001*	0.05 (0.02, 0.1)	< 0.0001*
Age	1.03 (1.01, 1.04)	< 0.0001*	1.03 (1.02, 1.04)	< 0.0001*
Sex—female	0.77 (0.64, 0.92)	0.0079*	0.82 (0.69, 0.98)	0.0344*
**Race/ethnicity**				
Black/African American	0.58 (0.38, 0.89)	0.0176*	0.54 (0.38, 0.77)	0.0014*
Another race/ethnicity	0.78 (0.45, 1.3)	0.3696	0.79 (0.48, 1.3)	0.3505
Self-reported health	1.04 (0.95, 1.1)	0.4048		
Ever smoked—yes	1.4 (1.1, 1.6)	0.0013*	1.4 (1.2, 1.6)	0.0009*
Percent non-Hispanic white	1.2 (0.85, 1.7)	0.3291		
Covered by Medicare—yes	1.1 (0.86, 1.5)	0.3799		
Number of times respondent spent night in the hospital	1.2 (1.0, 1.3)	0.0066*	1.1 (1.0, 1.2)	0.0040*
Seen dentist in previous 2 years—yes	0.56 (0.45, 0.68)	< 0.0001*	0.57 (0.46, 0.69)	< 0.0001*
Index of lifetime traumas before age 18	1.2 (1.1, 1.4)	0.0043*	1.2 (1.1, 1.4)	0.0029*
Index of stressful life events	0.81 (0.69, 0.96)	0.0179*	0.80 (0.69, 0.93)	0.0056*
Mean NO_2_ for September (µg/m^3^)	1.0 (0.97, 1.0)	0.9523		
Mean O_3_ for April (µg/m^3^)	0.99 (0.96, 1.0)	0.7827		
Mean O_3_ for May (µg/m^3^)	1.0 (0.99, 1.0)	0.2622		
Mean O_3_ for November (µg/m^3^)	1.0 (0.98, 1.0)	0.9529		
**High sensitivity C-reactive protein (mg/dL)**				
Between 1–3	1.1 (0.82, 1.4)	0.6810		
Greater than 3	0.94 (0.71, 1.2)	0.6638		

*hs-CRP* high sensitivity C-reactive protein, *OR* odds ratio, *CI* confidence interval.

*p < 0.05 as significant findings in the analysis.

Model 4 (Table 3), the logit model without the confounders, has an increased McFadden’s R^2^ of 0.07. Only age, sex, race/ethnicity, having ever smoked, the number of times the respondent spent the night in the hospital, whether the respondent saw the dentist in the last two years, the index of lifetime traumas before the age of 18, and the index of stressful life events are retained from Model 1. The direction of the estimates is unchanged from Model 1, and most odds ratios increased in magnitude. The k-NNDA accuracy for Model 4 is relatively similar to Models 1–3: 57%.

Schoenfeld residuals indicate the assumption of proportionality over age within the covariates was met. The Cox model identified many more predictors than the logit model, likely due to the outcome being measured over age (which has a strong relationship with TL). Significant predictors of shortened TL in Model 5 (Table 4) include being a high school graduate (as compared to not completing high school), higher weight, lower cystatin C (which is likely due to advanced age^54^), not being covered by Medicare, having spent more nights in the hospital, and not having seen a dentist in the last two years. Further, having “a little” difficulty walking one block compared to no trouble, yielded a lower hazard of shortened TL. The remaining variables reported in Model 5 are confounders. With hs-CRP added to the model (Models 6 and 7, Tables 4 and 5), neither the binary or tri-level variable is significant. The accuracy from k-NNDA is little more than chance at 56.14% for the base Cox model (Model 5), 58.06% for binary hs-CRP (Model 6), and 58.27% for tri-level hs-CRP (Model 7).

**Table 4:** Hazard ratios from Cox proportional hazards model of short telomere length.

Variable	Model 5: Without hs-CRP		Model 6: With binary hs-CRP	
	HR (95% CI)	*p*	HR (95% CI)	*p*
Sex—Female	0.95 (0.77, 1.17)	0.6424	0.86 (0.68, 1.08)	0.1879
**Race/ethnicity**				
Black/African American	0.77 (0.52, 1.14)	0.1882	0.77 (0.5, 1.17)	0.2200
Another race/ethnicity	1.43 (0.9, 2.29)	0.1340	1.53 (0.94, 2.51)	0.0890
**Education level**				
GED	1.13 (0.75, 1.72)	0.5537	1.14 (0.73, 1.79)	0.5595
High school grad	1.54 (1.19, 2)	0.0012*	1.59 (1.2, 2.11)	0.0013*
Some college	1.23 (0.84, 1.79)	0.2932	1.22 (0.83, 1.79)	0.3128
College or more	1.13 (0.78, 1.63)	0.5237	1.14 (0.76, 1.72)	0.5314
Number of children ever born	0.99 (0.94, 1.04)	0.5957	0.99 (0.93, 1.04)	0.6112
Total household income	1 (1, 1)	0.9647	1 (1, 1)	0.9983
Self-reported health	1.08 (0.95, 1.23)	0.2292	1.09 (0.95, 1.24)	0.2295
Weight (kg)	1.01 (1.01, 1.02)	< 0.0001*	1.01 (1, 1.02)	0.0005*
Heart problems—yes	0.9 (0.73, 1.12)	0.3469	0.89 (0.71, 1.11)	0.2902
Arthritis—yes	0.92 (0.74, 1.14)	0.4427	0.97 (0.77, 1.23)	0.8086
**Stroke**				
Yes	0.75 (0.55, 1.03)	0.0750	0.76 (0.53, 1.07)	0.1163
TIA/possible stroke	1.58 (0.69, 3.6)	0.2781	1.81 (0.84, 3.88)	0.1284
NHANES equivalent HDL (mg/dL)	1 (0.99, 1.01)	0.6553	1 (1, 1.01)	0.6594
NHANES equivalent Cystatin C (mg/L)	0.62 (0.46, 0.83)	0.0014*	0.56 (0.39, 0.79)	0.0009*
Covered by Medicare—yes	0.12 (0.1, 0.16)	< 0.0001*	0.12 (0.09, 0.16)	< 0.0001*
Number of times respondent spent night in the hospital	1.15 (1.11, 1.19)	< 0.0001*	1.14 (1.03, 1.28)	0.0160*
Number of nursing home stays	1.12 (0.86, 1.46)	0.4125	1.32 (1, 1.72)	0.0466*
Seen dentist in previous 2 years—yes	0.63 (0.52, 0.76)	< 0.0001*	0.62 (0.51, 0.76)	< 0.0001*
Used home health services in previous 2 years—yes	0.86 (0.65, 1.14)	0.2841	0.76 (0.56, 1.02)	0.0645
Negative family social support	1.1 (0.95, 1.28)	0.1830	1.13 (0.97, 1.32)	0.1222
Negative friend social support	0.93 (0.74, 1.17)	0.5245	0.93 (0.72, 1.19)	0.5512
Index of fine motor skills	1.07 (0.9, 1.27)	0.4668	1.07 (0.88, 1.29)	0.5071
**Difficulty walking 1 block**				
Yes, a little	0.73 (0.59, 0.9)	0.0036*	0.72 (0.56, 0.92)	0.0073*
Yes, a lot	1.06 (0.43, 2.59)	0.9046	1.06 (0.33, 3.46)	0.9206
Percent non-Hispanic White	1.09 (0.43, 2.79)	0.8547	0.96 (0.35, 2.66)	0.9363
Percent without high school degree of population aged 25 +	0.32 (0.02, 4.54)	0.4016	0.18 (0.01, 3.79)	0.2699
Percent population in poverty	5.77 (0.58, 57.46)	0.1351	6.18 (0.52, 73.15)	0.1488
Percent in the county with a high school diploma	0.74 (0.13, 4.18)	0.7377	0.68 (0.12, 3.94)	0.6640
Population density per square mile	1 (1, 1)	0.4559	1 (1, 1)	0.4962
Percent of individuals identifying as Hispanic or Latina/o	1.01 (0.33, 3.08)	0.9928	1.16 (0.35, 3.8)	0.8072
Individual health rate review-0.5	0.91 (0.61, 1.35)	0.6334	0.9 (0.56, 1.46)	0.6814
Individual health rate review-1	1.08 (0.85, 1.37)	0.5295	1.1 (0.86, 1.41)	0.4324
Mean O_3_ for second quarter (April–June) (µg/m^3^)	1.01 (0.99, 1.02)	0.4368	1.01 (0.99, 1.02)	0.4338
Mean O_3_ for fourth quarter (October–December) (µg/m^3^)	1.01 (0.98, 1.04)	0.4978	1.01 (0.98, 1.04)	0.6864
Mean PM_2.5_ for first quarter (January–March) (µg/m^3^)	1 (0.95, 1.06)	0.9848	1 (0.94, 1.06)	0.9236
Mean PM_2.5_ for fourth quarter (October–December) (µg/m^3^)	0.99 (0.94, 1.03)	0.5978	0.98 (0.93, 1.03)	0.4477
**High sensitivity C-reactive protein (CRPbi) (mg/dL)**				
Greater than 3			1.19 (0.96, 1.47)	0.1167

*hs-CRP* high sensitivity C-reactive protein, *HR* hazards ratio, *CI* confidence interval; *N* = 3084 for cox proportional hazards model without hs-CRP, N = 2,813 for cox proportional hazards model with binary hs-CRP.

*p < 0.05 as significant findings in the analysis.

**Table 5:** Hazard ratios from Cox proportional hazards model of short telomere length.

Variable	Model 7: With tri-level hs-CRP		Model 8: Without Cofounders	
	HR (95% CI)	*p*	HR (95% CI)	*p*
Sex—Female	0.85 (0.68, 1.08)	0.1848		
**Race/ethnicity**				
Black/African American	0.77 (0.5, 1.17)	0.2191		
Another race/ethnicity	1.54 (0.94, 2.51)	0.0879		
**Education level**				
GED	1.14 (0.73, 1.79)	0.5601	1.5 (1.1, 2.1)	0.0132*
High school grad	1.59 (1.2, 2.11)	0.0014*	1.4 (1.2, 1.7)	0.0011*
Some college	1.22 (0.83, 1.79)	0.3108	1.2 (1.0, 1.5)	0.1607
College or more	1.14 (0.75, 1.74)	0.5338	1.2 (0.97, 1.4)	0.2130
Number of children ever born	0.99 (0.93, 1.04)	0.6122		
Total household income	1 (1, 1)	0.9974		
Self-reported health	1.09 (0.95, 1.24)	0.2299		
Weight (kg)	1.01 (1, 1.02)	0.0005*	1.01 (1.01, 1.4)	< 0.0001*
Heart problems—yes	0.89 (0.71, 1.11)	0.2858		
Arthritis—yes	0.97 (0.77, 1.23)	0.8119		
**Stroke**				
Yes	0.76 (0.53, 1.07)	0.1171		
TIA/possible stroke	1.81 (0.85, 3.85)	0.1221		
NHANES equivalent HDL (mg/dL)	1 (1, 1.01)	0.6566		
NHANES equivalent Cystatin C (mg/L)	0.56 (0.39, 0.78)	0.0008*		
Covered by Medicare—yes	0.12 (0.09, 0.16)	< 0.0001*	0.12 (0.10, 0.15)	< 0.0001*
Number of times respondent spent night in the hospital	1.14 (1.02, 1.28)	0.0174*	1.1 (1.1, 1.2)	< 0.0001*
Number of nursing home stays	1.32 (1, 1.73)	0.0487*		
Seen dentist in previous 2 years—yes	0.62 (0.51, 0.76)	< 0.0001*	0.57 (0.46, 0.69)	< 0.0001*
Used home health services in previous 2 years—yes	0.76 (0.56, 1.02)	0.0714	0.78 (0.63, 0.97)	0.0253*
Negative family social support	1.13 (0.97, 1.32)	0.1221		
Negative friend social support	0.93 (0.72, 1.19)	0.5527		
Index of fine motor skills	1.07 (0.88, 1.29)	0.5109		
**Difficulty walking 1 block**				
Yes, a little	0.72 (0.56, 0.91)	0.0072*	0.67 (0.56, 0.80)	< 0.0001*
Yes, a lot	1.06 (0.32, 3.47)	0.9258	0.73 (0.43, 1.2)	0.2328
Percent non-Hispanic White	0.96 (0.34, 2.68)	0.9342		
Percent without high school degree of population aged 25 +	0.18 (0.01, 3.8)	0.2698		
Percent population in poverty	6.18 (0.52, 73.15)	0.1485		
Percent in the county with a high school diploma	0.68 (0.12, 3.94)	0.6644		
Population density per square mile	1 (1, 1)	0.4953		
Percent of individuals identifying as Hispanic or Latina/o	1.16 (0.35, 3.8)	0.8086		
Individual health rate review-0.5	0.9 (0.56, 1.46)	0.6785		
Individual health rate review-1	1.1 (0.86, 1.41)	0.4330		
Mean O_3_ for second quarter (April–June) (µg/m^3^)	1.01 (0.99, 1.02)	0.4355		
Mean O_3_ for fourth quarter (October–December) (µg/m^3^)	1.01 (0.98, 1.04)	0.6852		
Mean PM_2.5_ for first quarter (January–March) (µg/m^3^)	1 (0.94, 1.06)	0.9260		
Mean PM_2.5_ for fourth quarter (October–December) (µg/m^3^)	0.98 (0.93, 1.03)	0.4489		
**High sensitivity C-reactive protein (CRPtri) (mg/dL)**				
Between 1–3	1.01 (0.79, 1.3)	0.9294		
Greater than 3	1.2 (0.92, 1.55)	0.1738		

*hs-CRP* high sensitivity C-reactive protein, *HR* hazards ratio, *CI* confidence interval; N = 2,813 cox proportional hazards model with tri-level hs-CRP.

*p < 0.05 as significant findings in the analysis.

Model 8 (Table 5), the Cox model without the confounders, is comprised of education, weight, Medicare coverage, the number of times the respondent spent the night in the hospital, whether the respondent saw the dentist in the last two years, whether the respondent used home health services in the last two years, and degree of difficulty of walking one block. The direction of the unbiased hazard ratios is unchanged in the model (compared to Model 5) for all variables/categories except the “Yes, a lot” response for whether the respondent had difficulty walking one block. The k-NNDA accuracy for Model 8 is relatively similar to Models 5–7: 55%. VIFs for all models did not exceed 10.

In Step 1 of mediation analysis, the predictors of TL have coefficients that are significantly different from zero (Table S3), thus, we progressed to Step 2 . The results from Step 2, estimating hs-CRP (Table S4), indicate that some of the model predictors have coefficients significantly different than zero. Therefore, we proceeded to Step 3, using hs-CRP to estimate TL, controlling for model predictors. In Step 3 (Table S5), the coefficient on hs-CRP is not significantly different from zero, and there are no noticeable reductions in other predictor coefficients. Therefore, there is no evidence in support of hs-CRP as a mediator in the logit models, and we do not complete Step 4. Like the logit models, the results of Step 1 (Table S6) and Step 2 (Table S7) for the Cox models justify continuation to the next step of the analysis. However, the results of Step 3 (Table S8) indicate a non-significant coefficient on hs-CRP and a lack of reduction in other predictor coefficients, indicating no support for hs-CRP as a mediator for the predictor variables and TL.

## Discussion

We set out to determine the roles (risk factor or confounder) of various demographic, health- and psychological-related, and contextual and environmental determinants of shortened TL among middle-older adults using HRS data. We also provided unbiased odds and hazard ratios. Using a social epidemiological model of chronic stress leading to increased inflammation, and thusly shortened TL (Fig. 1), we found limited evidence for our hypothesis that a broad range of social determinants of health affect TL via inflammation (as measured through hs-CRP) and conclude our Fig. 1 is invalidated. Instead, we uncovered some evidence of significant predictors from the domains explored, primarily detecting confounders, and no association with hs-CRP. This latter finding was not entirely unexpected given that some research notes a lack of relationship between hs-CRP and TL^55^, while other research points to a relationship^16^. Importantly, the mediation analysis did not indicate hs-CRP was a mediator of the relationship between the predictor variables and TL, suggesting it is not the mechanism through which exposure to stressors affects TL.

The results for demographic factors are consistent with prior literature: being a female is related to longer TL^19^, as is identifying as Black/African American^20^. Unsurprisingly, in our work, education does not display a linear relationship, with respondents who have a GED or are high school graduates having shorter telomeres in comparison to individuals without a high school education; higher education was previously associated with shorter TL^21^, longer TL^22^, etc. For health-related determinants, the link highlighted in our study between weight and TL may represent a confounding relationship; others have noted that body mass index is no longer significant in regression models when leptin is included, which points to leptin as a likely source of the relationship with TL^56^. Further, this study confirms prior research^23,56^ that respondents who reported smoking have higher odds of short TL; this is postulated to be due to a link between tobacco and TL.

The survival analysis demonstrated an age-dependent relationship between TL and Medicare where those insured through Medicare have longer TL. We did not control for whether respondents had additional medical insurance in the regression models as it did not change our predictor variable of interest by 10% or more. Therefore, this effect could be due to heterogeneity introduced by respondents who have multiple types of insurance, one of which is Medicare. In 2006, almost 97% of HRS respondents ages 65 and older were Medicare beneficiaries and approximately 58% of this age group also reported private insurance, indicating that having multiple sources of health insurance is common in this sample. Dual insurance status may have benefits for health by providing access to additional medical services. In light of the results here, though, more research is warranted to examine whether Medicare recipients reflect the greater American population for TL.

While the literature shows a lack of relationship between hospital stays and TL^25^, our study found a link, which is likely due to a much larger sample size. The finding of shorter TL in patients with an increased number of overnight hospital stays may be related to underlying frailty tied to shortened TL. Similarly, the relationship between having seen a dentist in the last two years and lower odds of short TL may indicate these respondents practice good dental hygiene and therefore did not experience (or had an early intervention with) periodontal disease, which is a condition that is associated with shortened TL through increased inflammation and oxidative stress^26^.

For psychological health, respondents reporting more trauma before age 18 have higher risk of short TL, consistent with other research^15,29^. The mechanism(s) by which early adversity affects TL may include dysregulated stress signaling and increased inflammation, among others^29^. In adulthood, stressful life events do not display a straightforward relationship to TL^57^, and may be a confounder to shorter TL. Childhood adversity is a significant predictor of shortened TL in models with both childhood and adult adversity^10^. In the logit model, we showed childhood adversity was associated with shorter TL, while stressful events in adulthood corresponded with longer TL. There is some evidence that in vertebrates there is a trade-off in energy allocation during growth in the face of adversity that negatively affects TL, and accelerated growth leads to increased oxidative stress^58^. It is therefore possible that early life events impact TL to a greater degree than in adulthood, which would only be picked up by the logit as it is not calculated over age (as in the Cox regression). It is also plausible the adults who experienced increased stressful events also had longer telomeres at birth^6^. Further, a recent study showed a negative correlation between age and stressful life events in the HRS^59^, confirming this is a trend in the data. However, retrospective reports of life events are subject to recall bias, especially in older adults^60^. Therefore, this finding should be treated with caution.

Mobility measures were generally not related to TL, which is not surprising given the lack of relationship between physical performance and TL^61^. However, having “a little” difficulty walking was associated with lower risk of shortened TL compared to having no difficulty. It is possible that “a little” difficulty walking one block is reflective of people who have underlying medical conditions they treat through walking, which improves their overall health, and therefore, reduces the likelihood of having shorter TL. There may also be heterogeneity in the sample of individuals reporting no difficulties with walking: some individuals may assume they have no difficulties, while others may know they have no difficulties. No environmental or contextual factors tested was a significant predictor of TL. Interestingly, air pollution was a confounder to shorter TL, which is not surprising given the highly specific nature of the results reported in the literature^18,30^.

Confounding appears to be widespread when analyzing a binary measure of TL as many variables across the domains were confounders. This implies that TL is robust to the effects of many social determinants and the accumulating costs and weathering hypotheses are operating in moderation. Instead, access to medical care, underlying frailty, and few inequalities in the greater social environment appear to be linked to shortened TL. These findings also point to health inequities in our sample, which are generalizable to the greater United States due to study design of the HRS, and support the call to address social determinants simultaneously with improvements in primary health care^62^.

The limitations of this research include this analysis being cross-sectional; it cannot measure changes over time or generations and can only inform on whether individuals with increased stress also have shorter telomeres. Additionally, the race/ethnicity and sex variables are measured as categorical with few categories/binary, due to survey design, and it is recognized that this does not represent the underlying continuums. Further, our outcome variable is binary, which is likely an oversimplification of a complex biological process. However, the dichotomization of the variable was a necessity in order to produce statistical models that satisfied their underlying assumptions. We were also limited as to which inflammatory biomarkers could be tested due to timing of the TL samples, but that does not negate the value of examining hs-CRP alone in our research.

Some reports have also discussed potential variability in TL measurements using qPCR for different tissue types. Sample storage conditions, sample processing, and use of DNA extraction kits have resulted in different TL measurements using qPCR specifically for blood samples^63^. For salivary or buccal samples no data is available on what qPCR factors could cause variability in TL measurements in those tissue types. However, blood, saliva, and buccal samples also contain differing number of cells and cell ratios of cell types, which is important to consider when cross comparing TL measurements of different tissue types^63^. This is a limitation in comparing salivary TL length data outcomes with previous study outcomes on TL length involving other tissue types.

As noted above, there may be unanticipated heterogeneity in some of the variables and/or recall bias related to past life events. Finally, the models explained only a small amount of variance in TL. Based on past literature e.g.,^52,53^, we suspect this is related to the inability to include certain variables known to be associated with TL, including a history of infectious disease^64^, paternal age at birth^65^, and genetic factors^53,66^.

In conclusion, literature has shown conflicting evidence that hs-CRP and multiple social determinants of health are associated with shortened TL. With knowledge of these previous findings and using data from the HRS on older adults, we hypothesized that demographic, health- and psychological-related, and contextual/environmental factors predict a chronic stress response that leads to shortened TL through increased systemic inflammation. We assessed our hypothesis using logit and Cox models with and without hs-CRP, and conducted a mediation analysis. Although we did not find a significant relationship between hs-CRP and TL, we did discover evidence of some of the tested factors being significant predictors. Additionally, many were actually cofounders to shortened TL without association to hs-CRP. Our findings strengthen the understanding of how social determinants play a role in telomere attrition by reinforcing findings in previous literature and extending insights regarding the relationship between stress-related biomarkers and TL.

## Supplementary Information

Supplementary Tables.

## Data Availability

Data are available through reasonable request through the Health and Retirement Study.
