# Author Correction: How social/environmental determinants and inflammation affect salivary telomere length among middle-older adults in the health and retirement study

**DOI:** 10.1038/s41598-022-14839-x

**Published:** 2022-06-17

**Authors:** Margaret Gough Courtney, Josephine Roberts, Kanya Godde

**Affiliations:** University of La Verne, 1950 Third St., La Verne, CA USA

Correction to: *Scientific reports* 10.1038/s41598-022-12742-z, published online 25 May 2022

The Supplementary Information file published with this Article contained an error, where Supplementary Table S9 was omitted. The original Supplementary Tables file is provided below.

This error has now been corrected in the Supplementary Information file that accompanies the original Article.

## Supplementary Information

Supplementary Information.

