# Supplementary material for: Author Correction: How social/environmental determinants and inflammation affect salivary telomere length among middle-older adults in the health and retirement study
Source: Sci Rep. 2022 Jun 17;12:10195. doi: 10.1038/s41598-022-14839-x (PMC9205894; doi:10.1038/s41598-022-14839-x)
Supplement: Supplementary file 1 — Supplementary Information. [file 41598_2022_14839_MOESM1_ESM.docx]

**Supplementary Information for How Social/Environmental Determinants and Inflammation Affect Salivary Telomere Length among Middle-Older Adults in the Health and Retirement Study**

Margaret Gough Courtney, PhD, Josephine Roberts, Kanya Godde, PhD

Valid Non-Zero Biomarker Weight (Ages: <=54 to 97+)

N= 4,950

Full Telomere Study Sample for 2008 (Ages: <=41 to 97+)

N= 5,519

Non-Missing on Predictor Variables (Ages: <=54 to 95+)

N= 4,206

Non-Missing on Predictor Variables and hs-CRP (Ages: <=54 to 95+)

N= 3,761

**Figure S1. Telomere Length Sample Flow Chart and Minimum and Maximum Age**

*Psychological Measures Explained*

Lifetime traumas before age 18 is a sum of the experience of four types of trauma occurring before age 18: repeating a year of school, getting in trouble with the police, having a parent who drank or used drugs so often that it became a problem for the family, and being physically abused by a parent. The index of stressful life events summarizes the experience of six types of stressful events occurring for the respondent in the past five years: involuntary job loss; being unemployed and looking for work for more than three months; having someone else in the household unemployed and looking for work for more than three months; moving to a worse residence or neighborhood; being robbed or having home burglarized; and being a victim of fraud. The negative social support variables for family and friends are indices derived from four questions asked about family and friends: 1) How often do they make too many demands on you? 2) How much do they criticize you? 3) How much do they let you down when you are counting on them? 4) How much do they get on your nerves? to which respondents can answer “a lot”, “some”, “a little”, or “not at all”. Higher scores on the index indicate more negative support.

**Table S1.**

Comparison of Full Sample to Logit Model Analytic Sample

|  | **Telomere Regression**  **Full Sample** | | | **Telomere Regression**  **Analytic Sample** | |
| --- | --- | --- | --- | --- | --- |
| **Variable** | **Mean (SD) Proportion** | **95% CI** | **N** | **Mean (SD) Proportion** | **95% CI** |
| Short telomere length |  |  | 4,950 |  |  |
| No | 0.76 (0.01) | (0.75, 0.78) |  | 0.76 (0.01) | (0.75, 0.78) |
| Yes | 0.24 (0.01) | (0.22, 0.25) |  | 0.24 (0.01) | (0.22, 0.25) |
| Age | 66.90 (0.21) | (66.47, 67.33) | 4,950 | 66.87 (0.20) | (66.46, 67.28) |
| Sex |  |  | 4,950 |  |  |
| Male | 0.46 (0.01) | (0.44, 0.47) |  | 0.46 (0.01) | (0.45, 0.48) |
| Female | 0.54 (0.01) | (0.53, 0.56) |  | 0.54 (0.01) | (0.52, 0.55) |
| Race/Ethnicity |  |  | 4,950 |  |  |
| White | 0.87 (0.01) | (0.85, 0.88) |  | 0.88 (0.01) | (0.86, 0.90) |
| Black/African American | 0.09 (0.01) | (0.08, 0.10) |  | 0.08 (0.01) | (0.07, 0.10) |
| Another race/ethnicity | 0.04 (0.01) | (0.04, 0.06) |  | 0.04 (0.00) | (0.03, 0.05) |
| Ever smoked |  |  | 4,915 |  |  |
| No | 0.42 (0.01) | (0.41, 0.4) |  | 0.43 (0.01) | (0.41 0.45) |
| Yes | 0.58 (0.01) | (0.56, 0.59) |  | 0.57 (0.01) | (0.55, 0.59) |
| Number of times respondent spent night in the hospital | 0.42 (0.02) | (0.38, 0.46) | 4,934 | 0.42 (0.02) | (0.38, 0.46) |
| Seen dentist in previous 2 years |  |  | 4,948 |  |  |
| No | 0.34 (0.01) | (0.31, 0.36) |  | 0.33 (0.01) | (0.30, 0.35) |
| Yes | 0.66 (0.01) | (0.64, 0.69) |  | 0.67 (0.01) | (0.65, 0.70) |
| Index of lifetime traumas before age 18 | 0.47 (0.02) | (0.44, 0.50) | 4,437 | 0.47 (0.02) | (0.44, 0.50) |
| Index of stressful life events | 0.33 (0.014) | (0.31, 0.36) | 4,389 | 0.33 (0.01) | (0.31, 0.36) |

*Note*: CI = confidence interval; SD = standard deviation; N = 4,295 for Telomere Regression Analytic Sample. Telomere length was dichotomized into yes or no for having short telomere length were the lowest 25% of the distribution has short telomere length and the rest has normal length.

**Table S2.**

Comparison of Full Sample to Cox Model Analytic Sample

|  | **Telomere Regression**  **Full Sample** | | | | **Telomere Regression**  **Analytical Sample** | |
| --- | --- | --- | --- | --- | --- | --- |
| **Variable** | **Mean (SD) Proportion** | **95% CI** | | **N** | **Mean (SD) Proportion** | **95% CI** |
| Short telomere length |  | |  | 4,950 |  |  |
| No | 0.76 (0.01) | | (0.75, 0.78) |  | 0.76 (0.01) | (0.75, 0.78) |
| Yes | 0.24 (0.01) | | (0.22, 0.25) |  | 0.24 (0.01) | (0.22, 0.25) |
| Age | 66.90 (0.21) | | (66.47, 67.33) | 4,950 | 66.83 (0.22) | (66.39, 67.27) |
| Weight | 80.84 (0.40) | | (80.04, 81.64) | 4,907 | 80.90 (0.40) | (80.10, 81.69) |
| Education |  | |  | 4,949 |  |  |
| Less than high school | 0.17 (0.01) | | (0.15, 0.19) |  | 0.16 (0.01) | (0.15, 0.18) |
| GED | 0.04 (0.00) | | (0.03, 0.05) |  | 0.04 (0.00) | (0.03, 0.05) |
| High school grad | 0.30 (0.01) | | (0.29, 0.32) |  | 0.30 (0.01) | (0.29, 0.32) |
| Some college | 0.24 (0.01) | | (0.23, 0.26) |  | 0.24 (0.01) | (0.23, 0.26) |
| College and above | 0.25 (0.01) | | (0.23, 0.27) |  | 0.25 (0.01) | (0.23, 0.27) |
| Walk 1 block |  | |  | 4,924 |  |  |
| No | 0.86 (0.01) | | (0.85, 0.87) |  | 0.86 (0.01) | (0.85, 0.87) |
| Yes, a little | 0.13 (0.01) | | (0.12, 0.14) |  | 0.13 (0.01) | (0.12, 0.14) |
| Yes, a lot | 0.01 (0.00) | | (0.01, 0.01) |  | 0.01 (0.00) | (0.01, 0.01) |
| Covered by Medicare |  | |  | 4,941 |  |  |
| No | 0.47 (0.01) | | (0.45, 0.49) |  | 0.47 (0.01) | (0.45, 0.49) |
| Yes | 0.53 (0.01) | | (0.5, 0.55) |  | 0.53 (0.01) | (0.51, 0.55) |
| Number of times respondent spent night in the hospital | 0.42 (0.02) | | (0.38, 0.46) | 4,934 | 0.42 (0.02) | (0.38, 0.46) |
| Seen dentist in previous 2 years |  | |  | 4,948 |  |  |
| No | 0.34 (0.01) | | (0.31, 0.36) |  | 0.33 (0.01) | (0.31, 0.36) |
| Yes | 0.66 (0.01) | | (0.64, 0.69) |  | 0.67 (0.01) | (0.64, 0.69) |
| Used home health services in previous 2 years |  | |  | 4,948 |  |  |
| No | 0.93 (0.00) | | (0.93, 0.94) |  | 0.93 (0.00) | (0.93, 0.94) |
| Yes | 0.07 (0.00) | | (0.06, 0.07) |  | 0.07 (0.00) | (0.06, 0.07) |

*Note*: CI = confidence interval; SD = standard deviation: N = 4,853 for Telomere Regression Analytic Sample. Telomere length was dichotomized into yes or no for having short telomere length were the lowest 25% of the distribution has short telomere length and the rest has normal length

**Table S3.**

Logit Model Mediation Assessment using STL as an outcome variable

Step 1: use model predictors to estimate telomere length and look for coefficients significantly different from zero

| **Variable** | **OR (SE)** | **p** | **95% CI** |
| --- | --- | --- | --- |
| Intercept | -2.99 (0.36) | <0.01* | (-3.72, -2.27) |
| Age | 0.03 (0.00) | <0.01* | (0.02, 0.04) |
| Sex – Female | -0.20 (0.09) | 0.03* | (-0.38, -0.02) |
| Race/ethnicity |  |  |  |
| Black/African American | -0.62 (0.18) | <0.01* | (-0.98, -0.25) |
| Another race/ethnicity | -0.24 (0.25) | 0.35 | ( -0.74, 0.27) |
| Ever smoked – yes | 0.32 (0.09) | <0.01* | (0.14, 0.50) |
| Number of times respondent  spent night in the hospital | 0.11 (0.04) | <0.01* | (0.04, 0.18) |
| Seen dentist in previous 2 years – yes | -0.57 (0.10) | <0.01* | (-0.77, -0.37) |
| Index of lifetime traumas before age 18 | 0.19 (0.06) | <0.01* | (0.07, 0.31) |
| Index of stressful life events | -0.22 (0.08) | 0.01* | (-0.37, -0.07) |

*Note*: OR = odds ratio; SE = standard error; CI = confidence interval; STL = short telomere length; N = 4,295.

*p<0.05 as significant findings in the analysis.

**Table S4.**

Logit Model Mediation Assessment using STL as an outcome variable

Step 2: use model predictors to estimate CRP and look for coefficients significantly different from zero

| **Variable** | **OR (SE)** | **p** | **95% CI** |
| --- | --- | --- | --- |
| Intercept | -0.37 (0.35) | 0.29 | (-1.08, 0.33) |
| Age | -0.01 (0.00) | 0.03* | (-0.02, -0.00) |
| Sex – Female | 0.36 (0.07) | <0.01* | (0.21, 0.51) |
| Race/ethnicity |  |  |  |
| Black/African American | 0.45 (0.15) | <0.01* | (0.16, 0.74) |
| Another race/ethnicity | 0.08 (0.23) | 0.73 | (-0.38, 0.55) |
| Ever smoked – yes | 0.27 (0.10) | 0.01* | (0.06, 0.47) |
| Number of times respondent  spent night in the hospital | 0.26 (0.05) | <0.01* | (0.16, 0.35) |
| Seen dentist in previous 2 years – yes |  |  |  |
| Index of lifetime traumas before age 18 | -0.01 (0.06) | 0.87 | (-0.13, 0.12) |
| Index of stressful life events | 0.03 (0.07) | 0.70 | (-0.11, 0.17) |

*Note*: OR = odds ratio; SE = standard error; CI = confidence interval; STL = short telomere length; N = 3,842.

*p<0.05 as significant findings in the analysis.

**Table S5.**

Logit Model Mediation Assessment using STL as an outcome variable

Step 3: Use CRP to predict telomere length, controlling for model predictors and look for CRP coefficient significantly different from zero and reduction in other predictor coefficients

| **Variable** | **OR (SE)** | **p** | **95% CI** |
| --- | --- | --- | --- |
| Intercept | -3.00 (0.37) | <0.01* | (-3.74, -2.26) |
| High sensitivity C-reactive protein |  |  |  |
| Less than or equal to 3 | -0.07 (0.10) | 0.50 | (-0.27, 0.13) |
| Age | 0.03 (0.00) | <0.01* | (0.02, 0.04) |
| Sex – Female | -0.29 (0.10) | <0.01* | (-0.49, -0.10) |
| Race/ethnicity |  |  |  |
| Black/African American | -0.58 (0.19) | <0.01* | (-0.96, -0.19) |
| Another race/ethnicity | -0.23 (0.27) | 0.39 | (-0.78, 0.31) |
| Ever smoked – yes | 0.35 (0.09) | <0.01* | (0.17, 0.54) |
| Number of times respondent  spent night in the hospital | 0.15 (0.05) | <0.01* | (0.05, 0.26) |
| Seen dentist in previous 2 years – yes | -0.62 (0.10) | <0.01* | (-0.82, -0.41) |

*Note*: OR = odds ratio; SE = standard error; CI = confidence interval; STL = short telomere length; N = 3,842.

*p<0.05 as significant findings in the analysis.

**Table S6.**

Cox Model Mediation Assessment using STL as an outcome variable

Step 1: use model predictors (but not CRP) to estimate short telomere length and look for coefficients significantly different from zero

| **Variable** | **HR (SE)** | **p** | **95% CI** |
| --- | --- | --- | --- |
| Weight | 1.01 (0.00) | <0.01* | (1.01, 1.02) |
| Education |  |  |  |
| GED | 1.52 (0.25) | 0.02* | (1.09, 2.12) |
| High school grad | 1.44 (0.16) | <0.01* | (1.15, 1.80) |
| Some college | 1.22 (0.17) | 0.16 | (0.92, 1.61) |
| College and above | 1.17 (0.15) | 0.23 | (0.90, 1.52) |
| Walk 1 block |  |  |  |
| Yes, a little | 0.68 (0.06) | <0.01* | (0.60, 0.80) |
| Yes, a lot | 0.73 (0.19) | 0.25 | (0.43, 1.24) |
| Covered by Medicare – yes | 0.12 (0.01) | <0.01* | (0.10, 0.15) |
| Number of times respondent spent night in the hospital | 1.12 (0.02) | <0.01* | (1.08, 1.17) |
| Seen dentist in previous 2 years – yes | 0.68 (0.05) | <0.01* | (0.60, 0.78) |
| Used home health services  in previous 2 years – yes | 0.78 (0.09) | 0.03* | (0.62, 0.97) |

*Note*: HR = hazards ratio; SE = standard error; CI = confidence interval; STL = short telomere length; N = 4,853.

*p<0.05 as significant findings in the analysis.

**Table S7.**

Cox Model Mediation Assessment using STL as an outcome variable (N = 4,853)

Step 2: use model predictors to estimate CRP and look for coefficients significantly different from zero

| **Variable** | **HR (SE)** | **p** | **95% CI** |
| --- | --- | --- | --- |
| Weight | 1.01 (0.00) | <0.01* | (1.01, 1.01) |
| Education |  |  |  |
| GED | 1.29 (0.13) | 0.02* | (1.05, 1.58) |
| High school grad | 1.14 (0.05) | 0.01* | (1.04, 1.26) |
| Some college | 1.15 (0.08) | 0.05* | (1.00, 1.31) |
| College and above | 1.10 (0.08) | 0.20 | (0.95, 1.28) |
| Walk 1 block |  |  |  |
| Yes, a little | 0.71 (0.04) | <0.01* | (0.63, 0.79) |
| Yes, a lot | 0.52 (0.13) | 0.01* | (0.31, 0.87) |
| Covered by Medicare – yes | 0.09 (0.01) | <0.01* | (0.08, 0.11) |
| Number of times respondent  spent night in the hospital | 0.98 (0.02) | 0.27 | (0.93, 1.02) |
| Seen dentist in previous 2 years – yes | 1.05 (0.05) | 0.28 | (0.96, 1.15) |
| Used home health services  in previous 2 years – yes | 0.77 (0.05) | <0.01* | (0.67, 0.87) |

*Note*: HR = hazards ratio; SE = standard error; CI = confidence interval; STL = short telomere length; N = 4,853.

*p<0.05 as significant findings in the analysis.

**Table S8.**

Cox Model Mediation Assessment using STL as an outcome variable (N = 4,337)

Step 3: Use CRP to predict telomere length, controlling for model predictors and look for coefficient of CRP that is significantly different from zero plus reduction in other predictor coefficients

| **Variable** | **HR (SE)** | **p** | **95% CI** |
| --- | --- | --- | --- |
| High sensitivity C-reactive protein |  |  |  |
| Less than or equal to 3 | 1.11 (0.10) | 0.23 | (0.93, 1.32) |
| Weight | 1.01 (0.00) | <0.01* | (1.01, 1.02) |
| Education |  |  |  |
| GED | 1.40 (0.26) | 0.08 | (0.96, 2.04) |
| High school grad | 1.30 (0.14) | 0.02* | (1.04, 1.61) |
| Some college | 1.01 (0.13) | 0.95 | (0.78, 1.30) |
| College and above | 0.94 (0.12) | 0.62 | (0.72, 1.22) |
| Walk 1 block |  |  |  |
| Yes, a little | 0.69 (0.06) | <0.01* | (0.57, 0.82) |
| Yes, a lot | 0.64 (0.25) | 0.25 | (0.30, 1.38) |
| Covered by Medicare – yes | 0.12 (0.01) | <0.01* | (0.10, 0.15) |
| Used home health services  in previous 2 years – yes | 0.80 (0.10) | 0.06 | (0.63, 1.01) |

*Note*: HR = hazards ratio; SE = standard error; CI = confidence interval; STL = short telomere length; N = 4,853.

*p<0.05 as significant findings in the analysis.
